# A thousand metagenome-assembled genomes of *Akkermansia* reveal phylogroups and geographical and functional variations in the human gut

**DOI:** 10.3389/fcimb.2022.957439

**Published:** 2022-08-02

**Authors:** Qing-Bo Lv, Shenghui Li, Yue Zhang, Ruochun Guo, Yan-Chun Wang, Yongzheng Peng, Xiao-Xuan Zhang

**Affiliations:** ^1^ College of Veterinary Medicine, Qingdao Agricultural University, Qingdao, China; ^2^ Puensum Genetech Institute, Wuhan, China; ^3^ College of Animal Science and Technology, Jilin Agricultural University, Changchun, China; ^4^ Department of Laboratory Medicine, Zhujiang Hospital, Southern Medical University, Guangzhou, China

**Keywords:** *Akkermansia muciniphila*, metagenome-assembled genome, population structure, geographical variation, functional specificity, gut microbiota

## Abstract

*Akkermansia muciniphila* has long been considered to be the only *Akkermansia* species in the human gut and has been extensively studied. The present study revealed the genomic architecture of *Akkermansia* in the human gut by analyzing 1,126 near-complete metagenome-assembled genomes, 84 publicly available genomes, and 1 newly sequenced *Akkermansia glycaniphila* strain from the human gut. We found that 1) the genomes of *Akkermansia* were clustered into four phylogroups with distinct interspecies similarity and different genomic characteristics and 2) *A. glycaniphila* GP37, a strain of *Akkermansia*, was isolated from the human gut, whereas previously, it had only been found in python. Amuc III was present in the Chinese population, and Amuc IV was mainly distributed in Western populations. A large number of gene functions, pathways, and carbohydrate-active enzymes were specifically associated with phylogroups. Our findings based on over a thousand genomes strengthened our previous knowledge and provided new insights into the population structure and ecology of *Akkermansia* in the human gut.

## Introduction

*Akkermansia* is a well-studied genus that has been regarded as a representative of the phylum Verrucomicrobia in the human and animal gut (Derrien et al., 2004; Derrien et al., 2010; Cani et al., 2022). To date, only two species of *Akkermansia*, *A. muciniphila* and *A. glycaniphila* (Derrien et al., 2004; Ouwerkerk et al., 2016), have been isolated and comprehensively described. *Akkermansia muciniphila* is widely present in the intestinal mucosa of human (Ley et al., 2008; Presley et al., 2010; Belzer and de Vos, 2012; Falony et al., 2016), and it can degrade the mucin in epithelial mucosa and produce diverse structural molecules such as short-chain fatty acids (Derrien et al., 2004; Derrien et al., 2010; Hagi and Belzer, 2021). The host range of the *Akkermansia* genus is wide, ranging from mammals (mainly *A. muciniphila*) to non-mammals (e.g., *A. glycaniphila* is isolated from python (Ouwerkerk et al., 2016)) that differed greatly in physiology, dietary structure, and composition of mucinous proteins in the gut (Ley et al., 2008). There is growing evidence showing that *A. muciniphila* is an excellent candidate probiotic. Previous studies have shown a health-promoting effect of *A. muciniphila* (Dao et al., 2016; Derrien et al., 2017; Zhai et al., 2019), owing to the negative correlation of the relative abundance of *A. muciniphila* in gut microbiota with multiple metabolic disorders, such as hyperlipidemia (Yu et al., 2021), severe obesity (Hasani et al., 2021), and type 2 diabetes (Pascale et al., 2019; Zhang et al., 2021). Furthermore, supplementation with *A. muciniphila* in mice exerted a protective effect on colitis induced by dextran sulfate sodium and prevented the age-related decline in the thickness of the colonic mucus layer (Bian et al., 2019; van der Lugt et al., 2019). In clinical trials, oral supplementation of *A. muciniphila* was considered a safe and well-tolerated intervention for weight loss, thus improving insulin sensitivity and reducing insulinemia and plasma total cholesterol (Depommier et al., 2019).

After investigating a large number of full-length 16S sequences in 2011, it had been shown that at least eight species of the *Akkermansia* genus reside in the human digestive tract (van Passel et al., 2011). However, only two strains, *A. muciniphila* ATCC BAA-835 and *A. glycaniphila* Pyt (van Passel et al., 2011; Ouwerkerk et al., 2017), were subjected to a whole-genomic sequencing until 2017. Therefore, we still need to expand our understanding of the distribution of *Akkermansia* in the population to improve their potential applications in biomedicine. In our previous study (Guo et al., 2017), we sequenced and analyzed the draft genomes for 39 A*. muciniphila* strains isolated from China, and the population structure of these species was divided into three phylogroups (Amuc I, II, and III). These phylogroups showed a high genetic diversity in metabolic and functional features. Recently, a large number of metagenome-assembled genomes (MAGs) of the human gut microbiome have been published, and these data provide an opportunity to characterize the genomes of some important bacteria (Pasolli et al., 2019; Almeida et al., 2021). Pasolli et al. characterized the human microbiome from different body parts, ages, and countries through a large number of MAGs (Pasolli et al., 2019). Karcher et al. reported five different *A. muciniphila* candidate species in the human gut using a large-scale population genome analysis of *Akkermansia* (Karcher et al., 2021). These studies have expanded our understanding of genomic variation and species diversity in *A. muciniphila*.

To our knowledge, *A. glycaniphila*, another member of *Akkermansia*, is a strain that has never been isolated in the human gut. *Akkermansia glycaniphila* also appears unable to be assembled in metagenomic data from the human gut microbiome. Here, we isolated an *A. glycaniphila* strain from the gut of a subject and sequenced its whole genome. This does not completely prove that *A. glycaniphila* is endemic in the human gut, but it does expand our understanding of the genus *Akkermansia*. In addition, we also comprehensively analyzed the geographical distribution characteristics of *Akkermansia* based on more than 1,000 published *Akkermansia* genomes. These results reinforce previous findings and provide new insights into *Akkermansia* research.

## Methods

### Quality control and genome sequencing

We included the MAGs of *Akkermansia* (**Supplement Table ST1**) from the data made public by two studies (Pasolli et al. (2019) and Kirmiz et al. (2020)). Isolated genomes were downloaded from the National Center of Biotechnology Information (NCBI) database (**Supplement Table ST2**). The source information of these MAGs and genomes was also collected, such as host, country, etc. Each MAG met the quality standard of completeness of more than 90% and contamination of less than 5%, estimated based on the CheckM lineage (Parks et al., 2015). An *A. glycaniphila* strain (GP37) was isolated from human feces that was primarily isolated as part of a previous study (Guo et al., 2017). Genomes were sequenced using the Illumina HiSeq2500 instrument, and genomic assembly of *A. glycaniphila* GP37 was performed based on the previous pipeline as described previously (Guo et al., 2017).

### Gene prediction and functional annotation

To unify the standards, a genome content prediction for all *Akkermansia* genomes was carried out using Prokka (v1.13.3) (Seemann, 2014). The coordinates of genomic features within sequences, including small rRNA (5S, 16S, and 23S rRNA), were identified by using RNAmmer (v1.2) (Lagesen et al., 2007). The protein-coding gene prediction was performed using Prodigal (Hyatt et al., 2010). 16S rRNA gene sequence similarity was calculated using BLAST+ (v2.9.0). The functional annotation of genes was based on the Kyoto Encyclopedia of Genes and Genomes (KEGG, downloaded in December 2020) (Kanehisa et al., 2021) and CAZy databases (dbCAN2 version, CAZyDB.07312020) (Zhang et al., 2018) using USEARCH (Edgar, 2010) and DIAMOND (Buchfink et al., 2015), respectively, with the parameters e-value <1e−10, identity >70%, and coverage percentage >70%.

### Bioinformatic analyses

A phylogenetic tree of the *Akkermansia* strains was constructed based on concatenated protein subsequences by PhyloPhlAn (v.0.99) (Segata et al., 2013) with default parameters. The phylogenetic tree was visualized using iTol (Letunic and Bork, 2016). Pairwise average nucleotide identity (ANI) between two genomes was calculated using FastANI (v1.1) (Jain et al., 2018). Statistical analyses were implemented on the R platform. Heatmap was performed using the “heatmap.2” function, and principal coordinates analysis (PCoA) was performed using the cmdscale function (*vegan* package) and visualized using the *ggplot2* package (Wickham, 2016). The BRIG software was used to visualize genome comparisons (Alikhan et al., 2011).

## Results and discussion

### Metagenome-assembled genomes and isolated genomes of *Akkermansia*

In order to decipher the population structure and geographical distribution of *Akkermansia*, a total of 1,126 *Akkermansia* MAGs conforming to the “near-complete” standard (completeness > 90% and contamination < 5%) from public data (Pasolli et al., 2019; Kirmiz et al., 2020) were included. Although metagenomic samples were widely collected from multiple human sites, almost all *Akkermansia* MAGs were detected from human fecal samples (**Supplement Table ST1**). This finding was in line with the previous studies showing that the gut, rather than other body sites, was a major habitat of *Akkermansia* (Geerlings et al., 2018). Similarly, only six non-*Akkermansia* Verrucomicrobia genomes were identified in a recent study reconstructing over 56,000 MAGs from the global human oral metagenomes (Zhu et al., 2021); this result also indicated a very low occurrence of *Akkermansia* in the human oral cavity.

**Figure 1:**
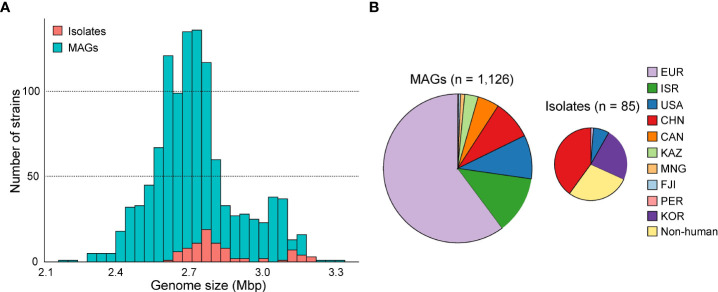
The genome size and country distribution of 1,211 strains of *Akkermansia*. **(A)** Distribution of genome size of 1,126 metagenome-assembled genomes (MAGs) and 85 isolated genomes. **(B)** Country distribution of 1,126 MAGs and 85 MAG isolated genomes.

The average completeness and contamination rates of 1,126 *Akkermansia* MAGs were 96.3% and 0.4%, respectively. The genomic data revealed varying genomic sizes ranging from 2.17 to 3.30 Mbp (average 2.73 Mbp, **Figure 1A**). The MAGs represented five continents and 22 different countries. The majority of the genomes (60.2%, 678/1,126) were from countries in Europe, and the others were from Israel (*n* = 141), the USA (*n* = 107), China (*n* = 97), Canada (*n* = 53), Kazakhstan (*n* = 33), Mongolia (*n* = 10), Fiji (*n* = 5), and Peru (*n* = 2) (**Figure 1B**). In view of the geographical and population spans and the integrity of 1,126 MAGs, we suggested that they effectively represented the characteristics of the human intestinal *Akkermansia* genus and could be used to answer fundamental questions regarding population structure and functional specificity of *Akkermansia*.

To extend the genomic content of *Akkermansia*, we also analyzed 84 isolated genomes from the NCBI database and one newly sequenced *Akkermansia* strain (GP37, an *A. glycaniphila* strain isolated from the human gut). The quality of these genomes was reassessed (**Supplement Table ST2**). The distribution of genome sizes for the isolated genomes was consistent with that of MAGs (**Figure 1B**). All of these strains were isolated from the feces, but their hosts were widely distributed, including humans (*n* = 61), mice (*n* = 13), chimpanzees (*n* = 3), and other animals (*n* = 8). Of the isolated genomes, 96.5% (82/85) were *A. muciniphila*, and the remaining three were *A. glycaniphila*.

### Population structure of *Akkermansia*

**Figure 2:**
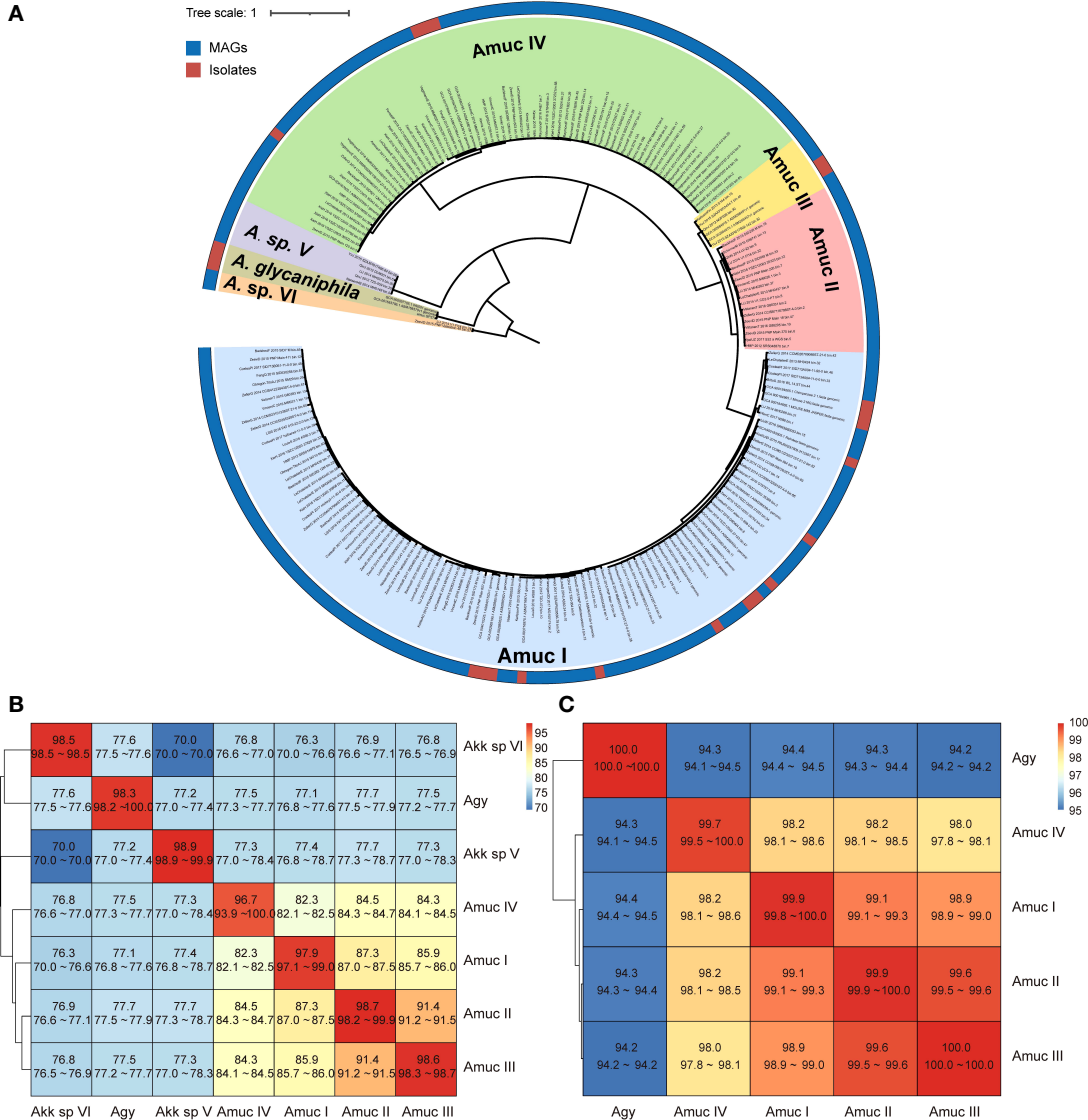
Phylogenetic analysis of *Akkermansia* genomes. **(A)** Phylogenetic tree of 1,126 MAG and 85 isolated genomes. Filling colors in the phylogenetic tree represent different species or phylogroups. The outer circle represents the original strains from MAGs and isolated genomes. For better visualization, only 10% of the strains of Amuc I and Amuc II were used, without changing the overall structure of the tree. **(B, C)** Heatmaps show the pairwise ANI among seven *Akkermansia* species and phylogroups **(B)** and the 16S sequence similarity among five *Akkermansia muciniphila* phylogroups and *Akkermansia glycaniphila* **(C)**.

The phylogenetic relationship of all 1,211 *Akkermansia* genomes was analyzed based on PhyloPhlAn, a method for improving the phylogenetic and taxonomic placement of microbes (Segata et al., 2013). We identified seven distinct phylogroups of *Akkermansia* (**Figure 2A**), including three *A. muciniphila* phylogroups (Amuc I, II, and III) reported by Guo et al. (2017) and a phylogroup of *A. glycaniphila*. The three *A. muciniphila* phylogroups accounted for 94% of all genomes, of which 945 were Amuc I, 181 were Amuc II, and 6 were Amuc III.

The clustering result of ANI on whole-genome data was identical to the phylogenetic analysis (**Figure S1**). The three known *A. muciniphila* phylogroups (Amuc I, II, and III) had average between-phylogroup ANIs ranging from 85% to 91% (**Figure 2B**), and the average 16S rRNA gene similarity was from 98.9% to 99.9% (**Figure 2C**). This finding suggested that these phylogroups were distinct subspecies, which was consistent with a previous study (Guo et al., 2017). In addition, the new phylogroup (containing 69 genomes) has an average ANI of 82%–84% with the three Amuc phylogroups I to III, and the average 16S rRNA gene similarity was 98.1%–98.5%. Therefore, this new phylogroup was defined as *A. muciniphila* subsp. IV (Amuc IV) according to the criterion for other *A. muciniphila* phylogroups (Guo et al., 2017; Kirmiz et al., 2020). *Akkermansia glycaniphila* and the two remaining new phylogroups showed a remarkable difference with regard to between-phylogroup ANI (<80%) and 16S rRNA gene similarity (<90%), suggesting that they were different species. These two branches were named as *Akkermansia* sp. V (containing five genomes) and *Akkermansia* sp. VI (containing two genomes). However, due to the small number of genomes and the lack of culture evidence, they cannot yet be defined as potential new species.

**Figure 3:**
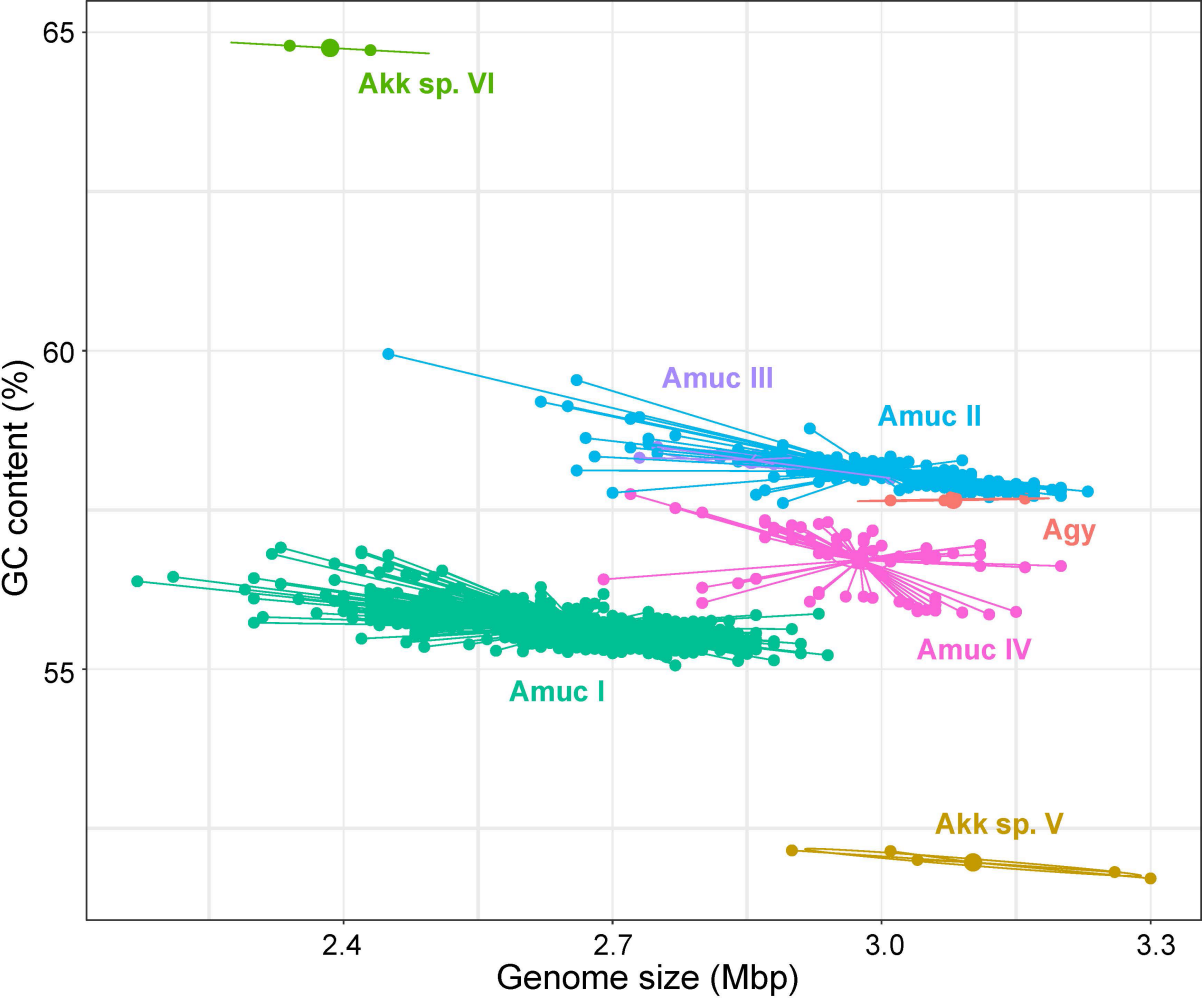
Scatter plot of the genome size and GC content of seven *Akkermansia* species and phylogroups.

There were significant differences in some genomic characteristics for the seven *Akkermansia* phylogroups. The strains of *A. glycaniphila* and *Akkermansia* sp. V had the largest genome size (average 3.08 and 3.10 Mbp, respectively; **Figure 3**), and the strains of *Akkermansia* sp. VI had the smallest genomes (average 2.39 Mbp). Among *A. muciniphila* subspecies, the genome sizes of Amuc II were the largest (average 2.99 Mbp), while Amuc I was the smallest (average 2.66 Mbp). The distribution of the number of proteins was consistent with that of genome sizes (**Figure S2**). The G+C content of *Akkermansia* sp. VI was extremely higher than the others (average 64.8%; **Figure 3**), while that of *Akkermansia* sp. V was remarkably lower (average 52.0%). Amuc II and III had a higher GC content (58.1% and 58.4%, respectively) compared with other *A. muciniphila* phylogroups, and Amuc I had the lowest GC content (55.7%). The representative genomes of Amuc I, II, III, and IV showed differences in several different genomic regions (**Figure S3**). Diversified genomic characteristics of the *Akkermansia* phylogroups suggested different evolution history and functional habits.

Belzer et al. (Belzer and de Vos, 2012) divided the *Akkermansia* phylogenetic tree into five clades according to the full-length 16S rRNA sequences. Among them, four clades contained human-associated sequences and one clade had a high diversity without human-derived sequences. In this study, we included the *Akkermansia* genomes from diverse transcontinental populations and found that the average similarity of 16S rRNA sequences between *A. muciniphila* and *A. glycaniphila* was 94.3%, and among Amuc I–IV phylogroups, it was >98%. This result indicated a relatively conservative 16S rRNA sequence in *Akkermansia* genomes, suggesting that more potential *Akkermansia* species or phylogroups are still undiscovered, especially in non-human animals. A recent study (Xing et al., 2019) constructed a phylogenetic tree based on 710 single-copy core genes shared by 23 *Akkermansia* genomes and divided *A. muciniphila* into four subspecies. The result was consistent with our findings showing that the genome of an *Akkermansia* strain (*Akkermansia* sp. KLE1797 from the NCBI database) belonged to a distinct phylogroup (in our study, Amuc IV). Amuc IV was also found by Kirmiz et al. based on 35 high-quality MAGs reconstructed from the feces of American children (Kirmiz et al., 2020). A comprehensive genomic diversity study reported that *Akkermansia* in the human gut can be divided into five candidate species (Karcher et al., 2021), of which the Amuc IV phylogroup was considered to contain two candidate species in this study. This is roughly the same as our findings.

### Global distribution of *Akkermansia* phylogroups

**Figure 4:**
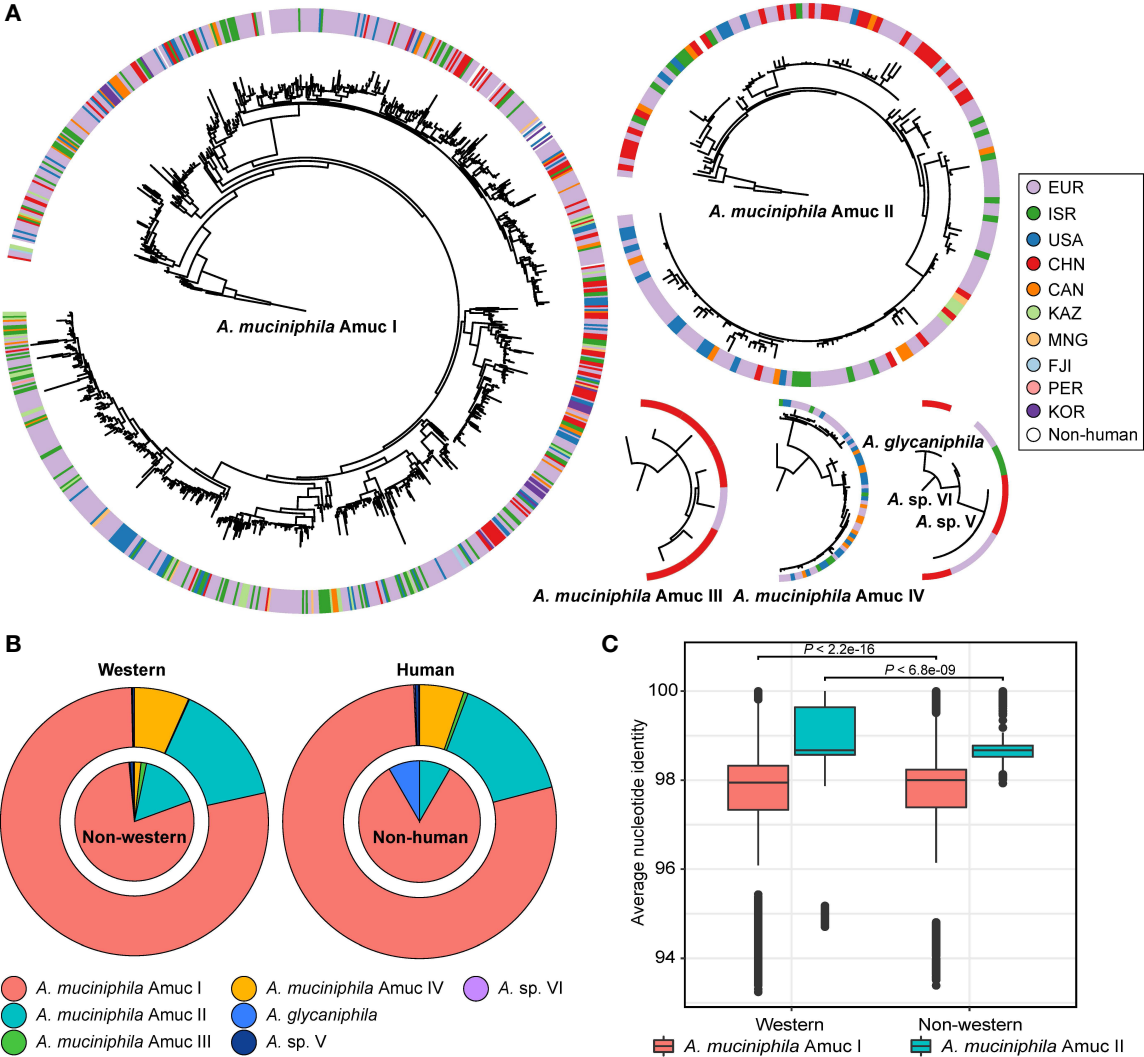
Geographic and host source of seven *Akkermansia* species and phylogroups. **(A)** Phylogenetic characteristics of *Akkermansia* species and phylogroups, with circles representing the national origin of each strain. **(B)** The distribution of *Akkermansia* species and phylogroups in Western and non-Western populations (left panel) and human and non-human hosts (right panel). **(C)** Boxplot shows the intra-phylogroup ANI comparisons between Western and non-Western strains.

The 1,211 *Akkermansia* genomes with wide distribution in 22 countries allowed us to investigate the biogeographical features of phylogroups. Also, to compare the differences between Western and non-Western populations, we define four Asian countries (China, Kazakhstan, Mongolia, and Israel) as non-Western and the rest as Western. We found that the two most dominant phylogroups, Amuc I and II, were extensively distributed globally (**Figures 4A, B**). Members of Amuc I and II were observed in trans-continental, trans-oceanic, cross-lifestyle populations and even appeared across-host considering that all non-human *A. muciniphila* isolates were placed in these two phylogroups. Amuc II, especially, had a higher intra-phylogroup genetic diversity in the Western populations compared to that of non-Western populations (**Figure 4C**). On the other hand, the geographic bias of Amuc III and IV was more prominent (**Figure 4A**; **Figure S4**). Moreover, 83.3% (five out of six) of the Amuc III genomes were from the gut microbiotas in a Chinese population and only one genome was from a European population. Conversely, all 69 genomes of Amuc IV were from Europe, the USA, Canada, and Israel, rather than from China or other countries. Moreover, the distributional modes of *Akkermansia* sp. V, *Akkermansia* sp. VI, and *A. glycaniphila* were still hard to accurately estimate due to the few numbers of genomes; however, all these species showed trans-continental distribution (**Figure S4**).

In recent years, the geographic deviation among different subspecies has been described in several gut bacteria such as *Prevotella copri* (Tett et al., 2019) and *Eubacterium rectale* (Karcher et al., 2020). These findings suggested that not only the geographical factor but also other unknown factors (e.g., diet difference between populations (Cheng et al., 2021; Díez-Sainz et al., 2022), or movement of individuals) are probably the forces for speciation of the gut bacteria. Similarly, except for Amuc III, Amuc I, II, and IV were observed in the gut microbiota of American children in the study of Kirmiz et al. (2020).

### Functional characteristics of *Akkermansia* phylogroups

**Figure 5:**
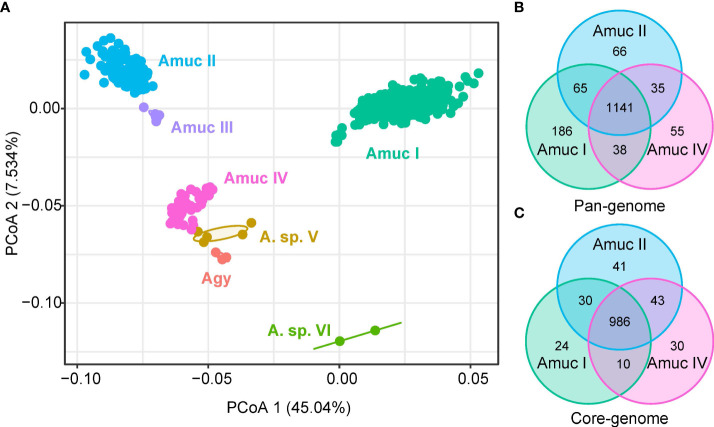
Comparison of KEGG functions among *Akkermansia* species and phylogroups. **(A)** PCoA analysis on the KO profiles of 1,211 strains. **(B, C)** Venn diagram shows the overlap of KOs in the pan-genome **(B)** and core-genome **(C)** of Amuc I, Amuc II, and Amuc IV.

It is possible that each *Akkermansia* phylogroup has a unique functional profile. To test this notion, we annotated all genomes using the KEGG database (Kanehisa et al., 2021) and identified a total of 1,740 KEGG orthologs (KOs). PCoA analysis based on the KO profiles revealed a clear separation among three major phylogroups (Amuc I, II, and IV; adonis *R* ^2^ > 0.25, *q* < 0.001 in pairwise comparison) (**Figure 5A**), while the functions of Amuc III strains were relatively close to Amuc II but were still significantly different (adonis *R* ^2^ = 0.058, *q* = 0.001). Likewise, the functions of *A. glycaniphila* and *Akkermansia* sp. V genomes were close to that of Amuc IV. We then compared the presence of KOs for three representative phylogroups (Amuc I, II, and IV) to identify the phylogroup-specific functions for them. In terms of pan-genome (KOs that occurred in at least one strain), the three phylogroups shared 1,141 functions, while 186, 66, and 55 functions especially occurred in Amuc I, II, and IV, respectively (**Figure 5B**). The Amuc I-specific functions were involved in the pathways of transporters (14 KOs), prokaryotic defense system (14 KOs), transcription factors (9 KOs), etc. (**Supplement Table ST3**), while the Amuc II-specific functions were related to transporters (6 KOs) and ABC transporters (6 KOs), peptidases and inhibitors (4 KOs), and others. The Amuc IV-specific functions were involved in pathways of transporters (6 KOs), two-component systems (4 KOs), peptidases and inhibitors (3 KOs), and others. In terms of core-genome (KOs that occurred in >90% of the strains), the three phylogroups shared 986 core functions, while 24, 41, and 30 functions especially occurred in Amuc I, II, and IV, respectively (**Figure 5C**). In terms of transport pathways, Amuc I-specific KOs mainly involve iron complex transporters, for example, K02016 and K02015. These substrate-binding proteins are usually involved in the transmembrane transport of iron, while Amuc IV-specific KOs participate in organic acid transporters, such as K08191. This is a hexuronate transporter, involved in the carbohydrate metabolic pathway of bacteria. These metabolic pathways are highly diverse in different species of bacteria (Rodionova et al., 2012). Therefore, the functions of these specific codes may suggest differences in energy utilization among different *Akkermansia* phylogroups. In other words, these phylogroup-specific functions were involved in multiple metabolism and transport pathways and potentially associated with the specific adaption mechanism for different *Akkermansia* phylogroups (**Supplement Table ST3**). In addition, functional differences between groups may be examples of the adaptive evolution of *Akkermansia*. For example, some subspecies lack the ability to make vitamin B_12_ (Karcher et al., 2021), which allows them to interact better with other species in the gut.

**Figure 6:**
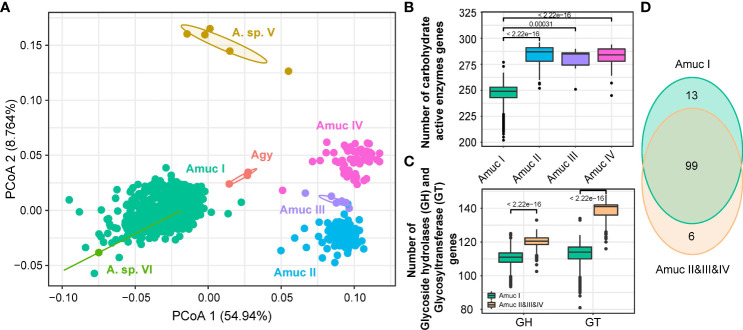
Comparison of CAZy functions among *Akkermansia* species and phylogroups. **(A)** PCoA analysis on the CAZymes families of 1,211 strains. **(B, C)** Boxplot showed the content comparison of CAZy enzyme-related genes annotated by the four phylogroups **(B)** and glycosyltransferase (GT) gene content between Amuc I and Amuc II–IV **(C)**; significance was calculated using the rank-sum test. **(D)** Venn diagram shows the overlap of CAZyme families between Amuc I and Amuc II–IV.

In order to further investigate the ability of carbohydrate formation and decomposition of *Akkermansia* species, the genomes were screened for carbohydrate-active enzymes (CAZymes) (Zhang et al., 2018). Notably, a remarkable separation was observed on the CAZyme profiles among members of Amuc I and II–IV (adonis *R* ^2^ = 0.56, *q* < 0.001; **Figure 6A**), while the strains of Amuc II, III, and IV were relatively closer. Compared to Amuc II–IV, strains of Amuc I had a fewer number of carbohydrate-active enzymes (*p* < 0.001; **Figure 6B**) and especially glycosyltransferase (GT) and glycoside hydrolase (GH) proteins (*p* < 0.001; **Figure 6C**). GT proteins are mostly related to protein glycosylation, cell wall polysaccharide synthesis, or synthesizing exopolysaccharides in the context of biofilm formation (Lairson et al., 2008). This may represent the adaptability of the strains of Amuc II–IV phylogroups to the synthesis of exopolysaccharides or other structural carbohydrates. Moreover, 13 CAZymes were specifically encoded in the pan-genome of Amuc I, while 6 CAZymes were specifically encoded in the genomes of Amuc II–IV members. Interestingly, glycosyl transferase family 61 (GT61, occurred in 98.6% of Amuc IV strains but none in others) and glycoside hydrolase family 130 (GH130, occurred in 78.3% of Amuc IV strains but none in others) were specifically encoded by Amuc IV (**Figure 6D**; **Supplement Table ST4**). The GT61 family involved in the synthesis of cell wall xylans is often reported in plant cells (Cenci et al., 2018). These MAGs mainly encode two enzymes (SVE69682.1 and SVE78114.1) of the GT61 family. Interestingly, this family is uncommon in prokaryotes, and as a result, it may indicate that the cell wall composition of Amuc IV members is different from that of other phylogroups. The isolation of this type of strain in future studies will help us further understand the role of these enzymes. Similarly, GH130 might potentially provide the ability of mannose hydrolysis for Amuc IV members (Saburi, 2016), and this phenomenon may be correlated to the geographical distribution differences of the Amuc IV phylogroup. In brief, the difference in functional profiles among *Akkermansia* phylogroups may have relativity to their ability for utilizing complex carbohydrates.

**Figure 7:**
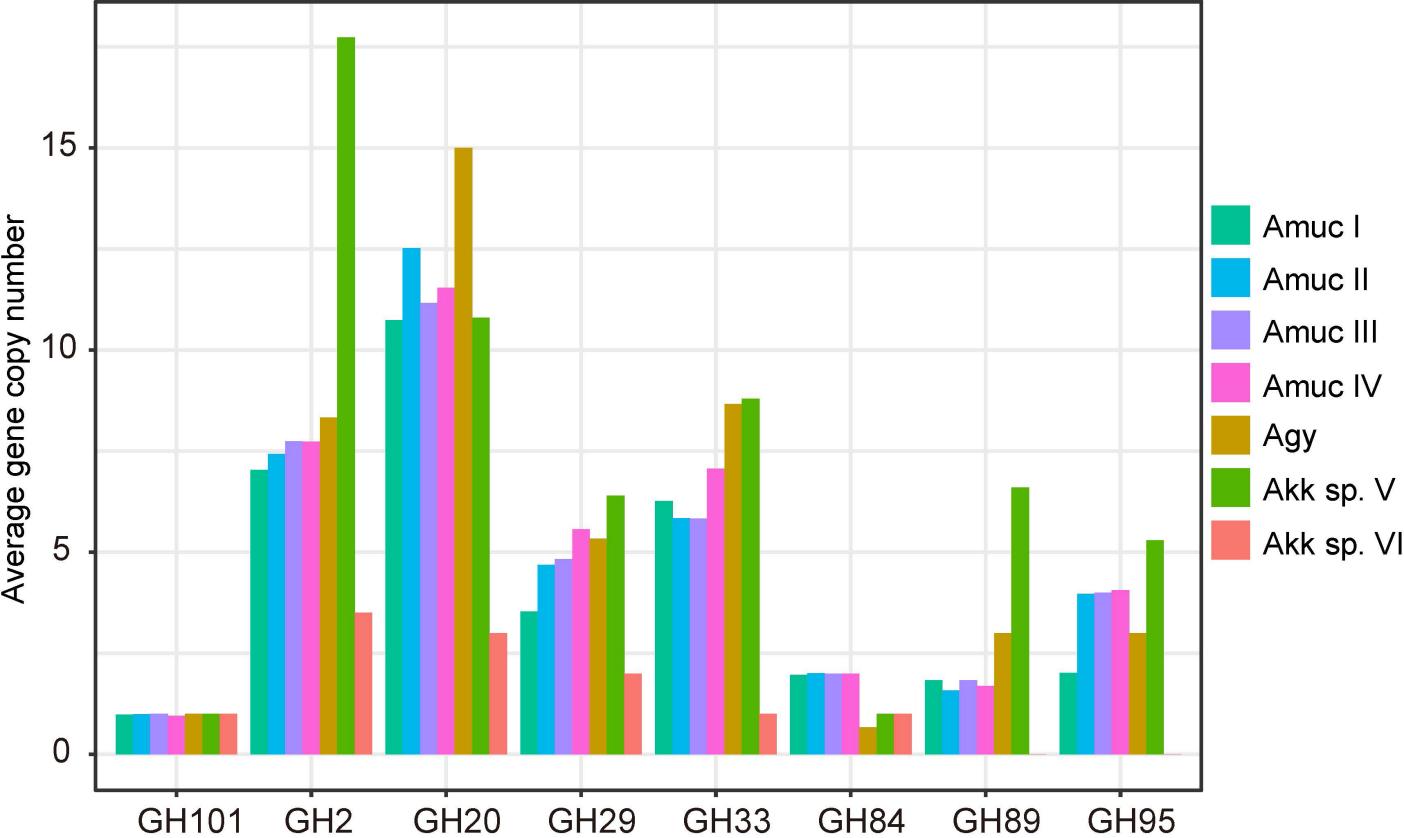
Average number of gene copies in each phylogroup of the eight mucin-degrading GH families.

In addition, we were concerned about the gene copy number of mucin-degrading GHs in each phylogroup. A total of eight mucin degradation-related GH families were annotated in all genomes (**Figure 7**). *Akkermansia* sp. VI has the least copy number compared to the other groups, and GH89 and GH95 were absent in these genomes. Compared with Amuc I, Amuc II, III, IV, *Akkermansia* sp. V, and Agy had higher gene copies in GH2, GH20, GH29, and GH95, most notably *Akkermansia* sp. V, which had relatively higher copy numbers on multiple mucin-associated GH. This difference may indicate the carbohydrate preference of each phylogroup strain. *Akkermansia* can degrade mucin into acetic acid and propionic acid, from which it can obtain energy (Kim et al., 2021). The diversity of mucin-related enzymes in *Akkermansia* may indicate that different groups metabolize mucin in different ways. However, we can provide only limited genomic evidence. In future studies, we need to obtain more culturable strains to test this hypothesis.

### Assembly of the genome of *Akkermansia glycaniphila* strain from human

GP37 is a strain of *A. glycaniphila* isolated and cultured from human feces. The genome assembly of this strain was assembled by whole-genome sequencing. Its genome size is 3.01 Mbp and has 2,554 genes, its GC content is 57.65%, its completeness is 94.56%, and its contamination rate is zero (**Supplement Table ST2**). The genome of GP37 contains 13 contigs and the N50 length is 663,974bp, which means that GP37 is a high-quality genome assembly. ANI is commonly used to describe the consistency between the genomes of strains and species. GP37 had greater than 98% ANI with other *A. glycaniphila* genomes (GCF_900097105.1_WK001 and GCF_001683795.1_ASM168379v1), suggesting that they are highly similar strains (**Figure S5**). In addition, GP37 encodes 270 predictive CAZymes, including 106 glycosyl transferases and 118 glycosyl hydrolases, indicating its role in energy metabolism.

## Conclusions

This study characterized the phylogeographic population structure and functional specificity of *Akkermansia* based on 1,126 near-complete MAGs and 85 isolated genomes. The *Akkermansia* genomes were placed into two previously isolated species (*A. muciniphila* and *A. glycaniphila*), and the *A. muciniphila* members were further divided into three previously described phylogroups (Amuc I, II, III, and IV). These species and phylogroups revealed a significant geographical distribution bias; especially, Amuc III was present in the Chinese population and Amuc IV was mainly distributed in Western populations. Functional analyses showed notable specificity in different *Akkermansia* species and phylogroups that were involved in some metabolism and transport pathways and in carbohydrate-active enzymes. In conclusion, our results showed that the *Akkermansia* members in the human gut had high genomic diversity and functional specificity and diverse geographical distribution characteristics.

## Data availability statement

The datasets presented in this study can be found in online repositories. The names of the repository/repositories and accession number(s) can be found below: https://www.ncbi.nlm.nih.gov/bioproject/PRJNA662466.

## Ethics statement

The studies involving human participants were reviewed and approved by the Ethics Committee of Zhujiang Hospital of Southern Medical University (2014-JYYXB-009). The patients/participants provided their written informed consent to participate in this study. Written informed consent was obtained from the individual(s) for the publication of any potentially identifiable images or data included in this article.

## Author contributions

X-XZ, YP, Q-BL, and SL conducted the study. Q-BL, SL, and YZ performed the bioinformatic analyses. X-XZ, Y-CW, YP, Q-BL, and SL wrote and edited the manuscript. All authors read and approved the final manuscript.

## Funding

This work was supported by the Research Foundation for Distinguished Scholars of Qingdao Agricultural University (665-1120044).

## Conflict of interest

The authors declare that the research was conducted in the absence of any commercial or financial relationships that could be construed as a potential conflict of interest.

## Publisher’s note

All claims expressed in this article are solely those of the authors and do not necessarily represent those of their affiliated organizations, or those of the publisher, the editors and the reviewers. Any product that may be evaluated in this article, or claim that may be made by its manufacturer, is not guaranteed or endorsed by the publisher.
