# Supplementary figures and images for: A thousand metagenome-assembled genomes of Akkermansia reveal phylogroups and geographical and functional variations in the human gut
Source: Front Cell Infect Microbiol. 2022 Aug 2;12:957439. doi: 10.3389/fcimb.2022.957439 (PMC9378777; doi:10.3389/fcimb.2022.957439)

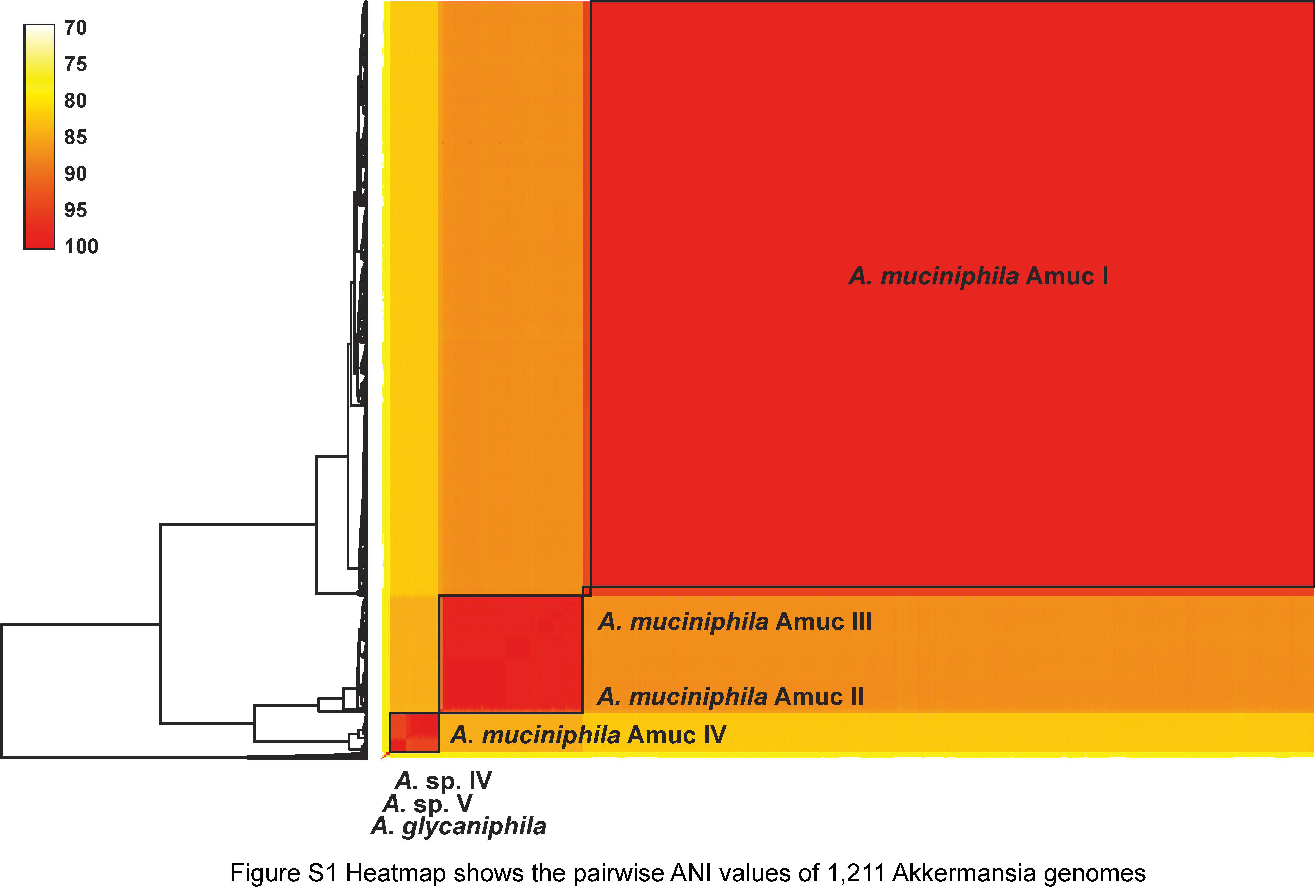

Supplement: Supplementary file 1 [file Image_1.tif]

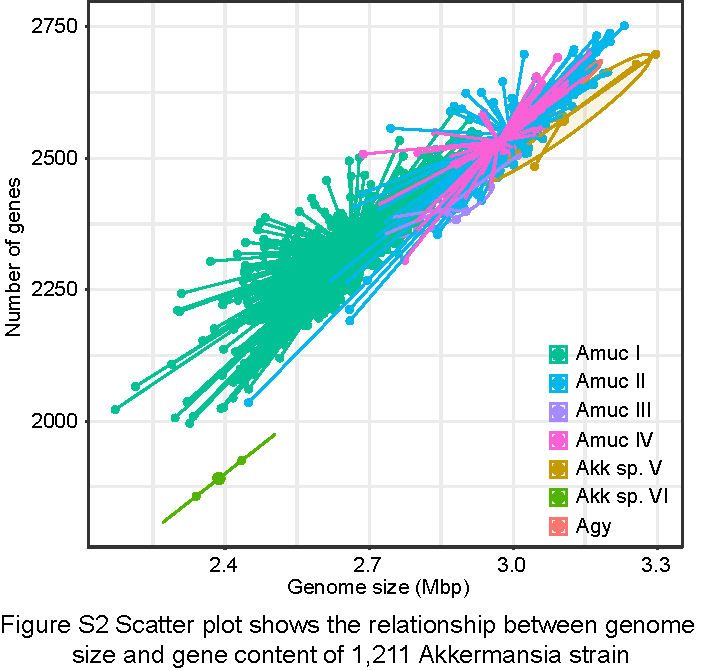

Supplement: Supplementary file 2 [file Image_2.tif]

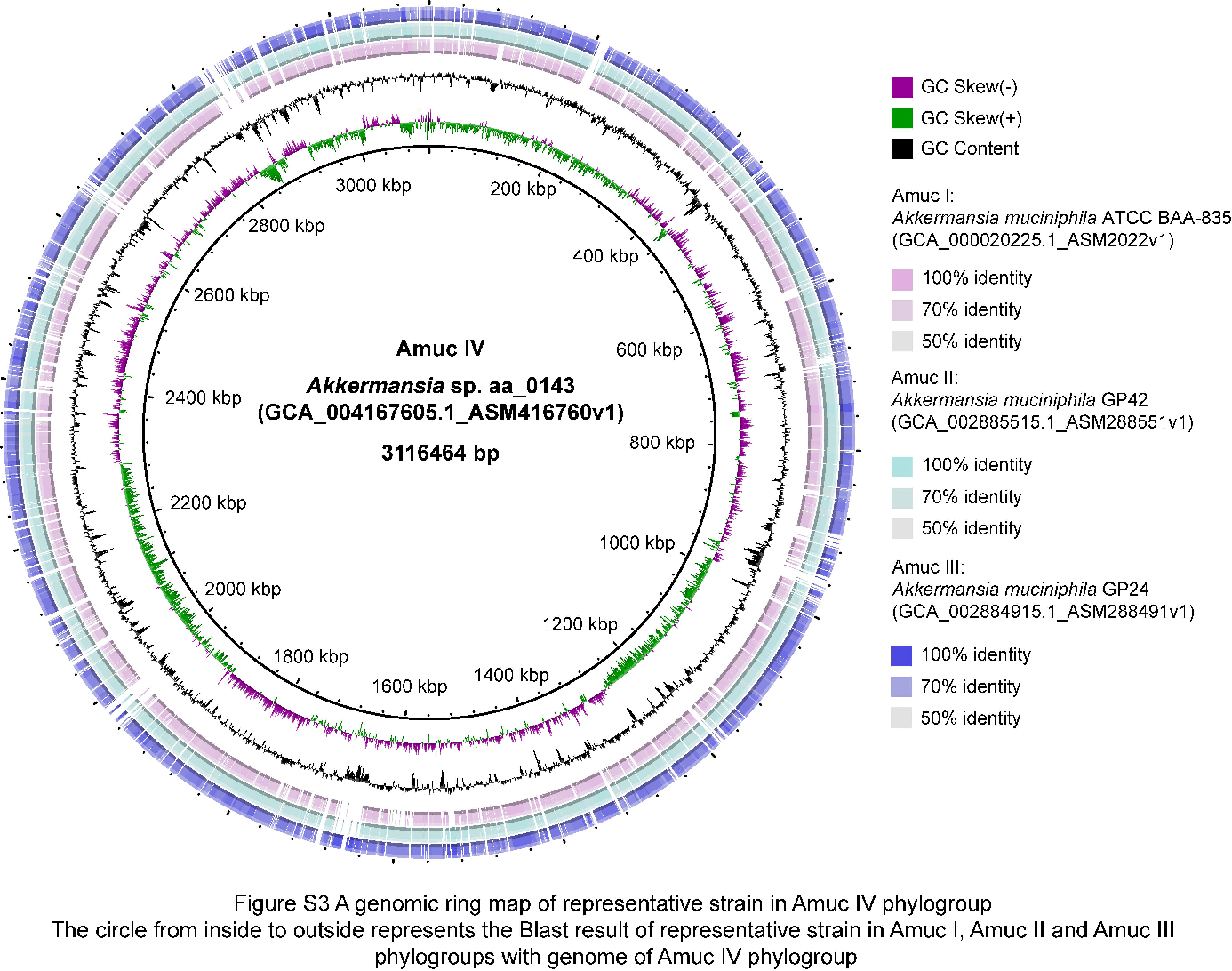

Supplement: Supplementary file 3 [file Image_3.tif]

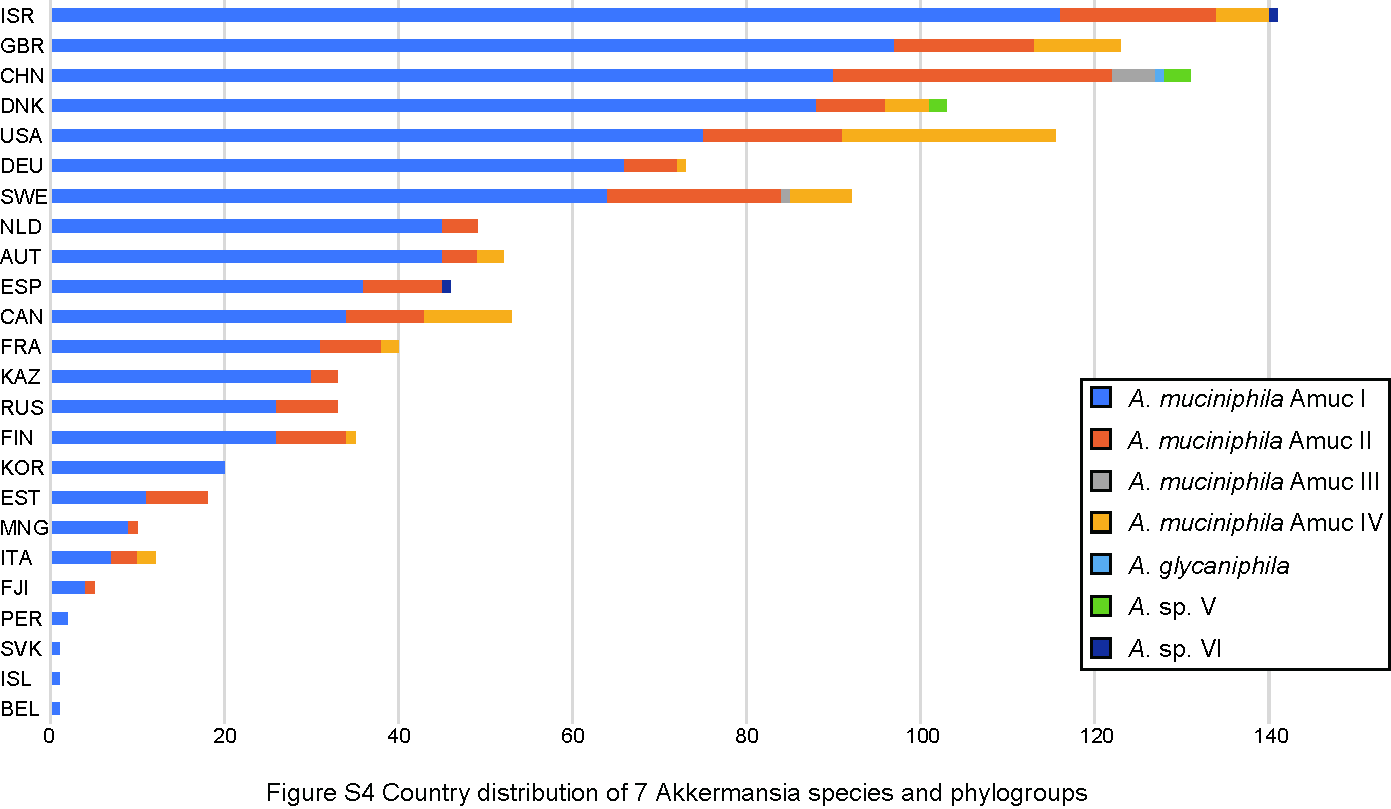

Supplement: Supplementary file 4 [file Image_4.tif]

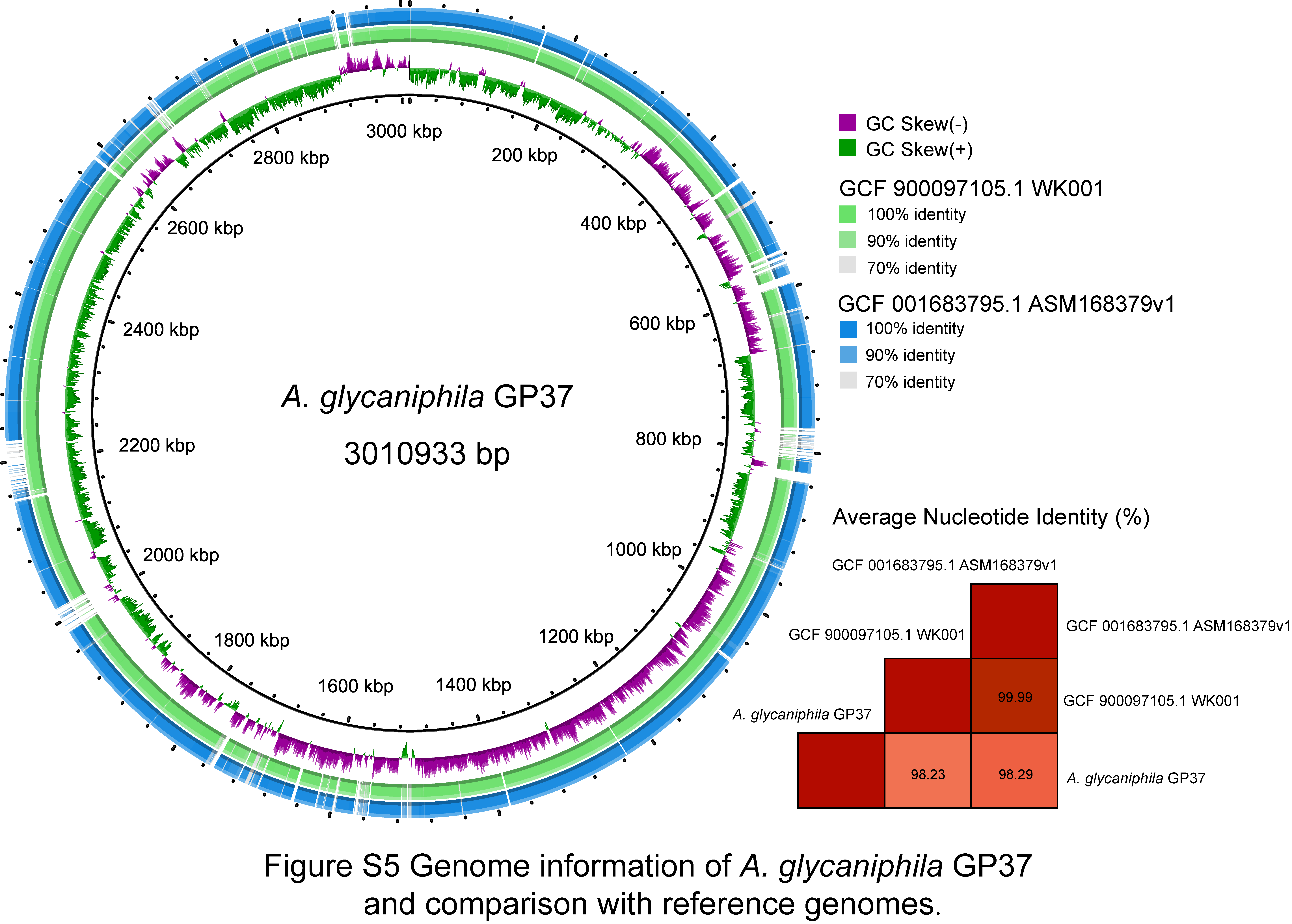

Supplement: Supplementary file 5 [file Image_5.tif]
